# Murine double minute 2 aggravates adipose tissue dysfunction through ubiquitin-mediated six-transmembrane epithelial antigen of prostate 4 degradation

**DOI:** 10.1016/j.isci.2022.104544

**Published:** 2022-06-07

**Authors:** Wei Zhao, Qiang Xu, Jiahui Yang, Xianghong Xie, Chunmei Li, Weihong Zhang, Enhui Chen, Yanfang Guo, Mingyue Gao, Jie Shi, Huabing Zhang, Hong Yao, Meixia Li, Li Yan, Fude Fang, Wenming Wu, Xiaojun Liu

**Affiliations:** 1 State Key Laboratory of Medical Molecular Biology, Institute of Basic Medical Sciences Chinese Academy of Medical Sciences & School of Basic Medicine Peking Union Medical College, Beijing 100005, China; 2 Department of General Surgery, Peking Union Medical College Hospital, Chinese Academy of Medical Science & Peking Union Medical College, Beijing 100730, China; 3 Department of Microbiology and Immunology, Shanxi Medical University, Taiyuan 030001, China; 4 Department of Pathophysiology, Institute of Basic Medical Sciences Chinese Academy of Medical Sciences & School of Basic Medicine Peking Union Medical College, Beijing 100005, China; 5 Department of Pathology, Peking Union Medical College Hospital, Chinese Academy of Medical Sciences and Peking Union Medical College, Beijing 100730, China; 6 Department of Biochemistry and Molecular Biology, School of Basic Medicine, Anhui Medical University, Hefei 230032, China; 7 State Key Laboratory of Brain and Cognitive Science, Institute of Biophysics, Chinese Academy of Sciences, Beijing 100101, China; 8 Department of General Surgery, State Key Laboratory of Complex Severe and Rare Diseases, Peking Union Medical College Hospital, Chinese Academy of Medical Science and Peking Union Medical College, Beijing 100730, China

**Keywords:** Biological sciences, Molecular biology, Immunology, Proteomics

## Abstract

Healthy adipose tissue is crucial to maintain normal energy homeostasis. Little is known about the role of murine double minute 2 (MDM2), an E3 ubiquitin ligase and has been highlighted in oncopathology, in adipose tissue. Our results indicated that MDM2 expression was associated with nutritional status. *Mdm2* adipocyte-specific knock-in (*Mdm2*-AKI) mice exhibited exacerbated weight gain, insulin resistance, and decreased energy expenditure. Meanwhile, chronic high-fat diet (HFD) exposure caused obvious epididymal white adipose tissue (eWAT) dysfunction, such as senescence, apoptosis, and chronic inflammation, thereby leading to hepatic steatosis in *Mdm2*-AKI mice. Mechanically, MDM2 could interact with six-transmembrane epithelial antigen of prostate 4 (STEAP4) and inhibit STEAP4 expression through ubiquitin-mediated STEAP4 degradation. Thereinto, the K18 and K161 sites of STEAP4 were ubiquitin-modificated by MDM2. Finally, STEAP4 restoration in eWAT of *Mdm2*-AKI mice on a HFD rescued MDM2-induced adipose dysfunction, insulin resistance, and hepatic steatosis. Summary, the MDM2-STEAP4 axis in eWAT plays an important role in maintaining healthy adipose tissue function and improving hepatic steatosis.

## Highlights

• Murine double minute 2 (MDM2) overexpression intensifies high-fat diet-induced adipose tissue dysfunction
• Adipocyte MDM2 overexpression aggravates insulin resistance and hepatosteatosis
• MDM2 decreases six-transmembrane epithelial antigen of prostate 4 (STEAP4) expression by ubiquitin-dependent STEAP4 degradation
• STEAP4 overexpression in eWAT alleviates MDM2-induced metabolic disorder

## Graphical abstract

**Figure:**
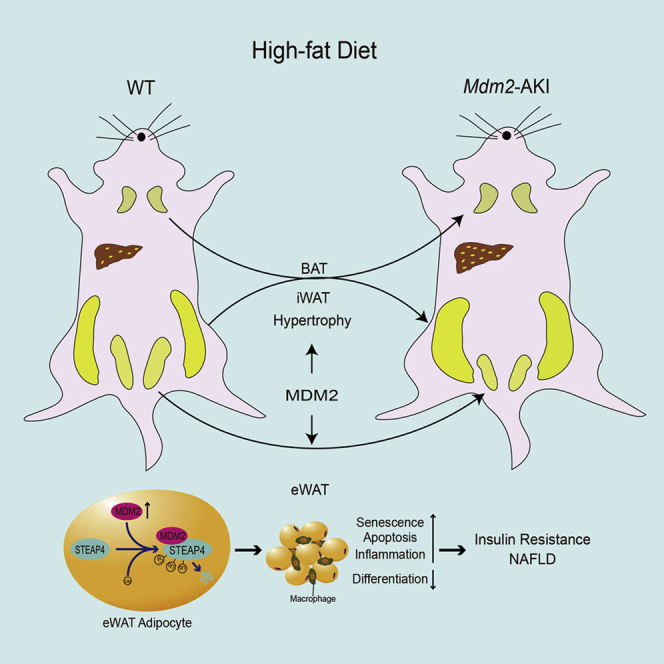

## Introduction

Adipose tissue is the master regulator of energy balance and nutritional homeostasis and its distribution and function change dramatically throughout life (Spiegelman and Flier, 2001; Tchkonia et al., 2010). Adipose tissue consists mainly of white adipose tissue (WAT), which is used to store energy, and brown adipose tissue (BAT), which produces heat to maintain body temperature. WAT can be anatomically categorized into visceral adipose tissue and subcutaneous adipose tissue. In mice, epididymal WAT (eWAT) and inguinal WAT (iWAT) are representative models of visceral and subcutaneous adipose tissues, respectively. Adipose tissue can primarily expand through the enlargement of existing adipocytes (hypertrophy) or through the differentiation of resident adipocyte precursors to form new adipocytes (hyperplasia). Hyperplasia of adipose tissue has generally been considered healthy and adaptive, whereas hypertrophy of adipocytes has been associated with adipose tissue dysfunction (Bluher, 2009; Ghaben and Scherer, 2019). Adipose tissue dysfunction has been associated with senescence, apoptosis, and inflammation. Many factors can induce cellular senescence in adipose tissue, thereby promoting impaired adipogenesis, failed sequestration of the senescence-associated secretory phenotype (SASP), such as lipotoxic fatty acids, inflammatory cytokine, and chemokine generation. These processes may reinforce each other and have systemic consequences, resulting in lipotoxicity in other metabolically sensitive organs, such as the liver and muscles, and aggravating insulin resistance (Tchkonia et al., 2010). In old age, adipose progenitor cells and preadipocytes dysdifferentiate and switch into a proinflammatory, senescent-like state (Tchkonia et al., 2010). A previous study indicated that eliminating senescent cells in adipose tissue could be a novel therapeutic target for treating obesity-induced metabolic dysfunction (Palmer et al., 2019). These senolytic interventions alleviated metabolic and adipose tissue dysfunction in obese mice, such as improving glucose tolerance and insulin sensitivity, lowering circulating inflammatory mediators, and promoting adipogenesis.

Murine double minute 2 (MDM2), an E3 ubiquitin ligase, has been highlighted in oncopathology as an upstream regulatory factor of the tumor suppressor p53 (Momand et al., 1992). Previous studies have indicated that p53 expression could be induced by a high-fat diet (HFD) in multiple tissues including adipose tissue, resulting in HFD-induced obesity and insulin resistance (Derdak et al., 2013; Minamino et al., 2009; Yokoyama et al., 2014). Pharmacological inhibitors of p53 can improve HFD-induced weight gain and hepatic steatosis (Derdak et al., 2013). As a key regulator of p53, MDM2 has also been shown to be involved in adipose metabolism. The study by Liu et al. showed that adipocyte-specific MDM2 deficiency triggered a series of aging-associated metabolic dysfunction dependent on p53 (Liu et al., 2018). Additionally, some studies have suggested that MDM2 is involved in the initiation of adipocyte differentiation via a p53-independent mechanism, such as inducing the expression of CCAAT/enhancer-binding protein δ and activating signal transducer and activator of transcription 3 (Hallenborg et al., 2012, 2016). A recent study demonstrated that MDM2 haploinsufficient in adipocytes induced overt obesity, glucose intolerance, and hepatic steatosis through a novel interplay with the transcriptional cofactors MORC Family CW-Type Zinc Finger 2 (MORC2) and LIPIN1 (Hallenborg et al., 2021). These studies suggested MDM2 has a crucial role in adipose tissue metabolism through p53-dependent and -independent mechanisms.

The six-transmembrane epithelial antigen of prostate 4 (STEAP4), also known as six-transmembrane protein of prostate 2 (STAMP2) or tumor necrosis factor-α (TNF-α) induced adipose-related protein (TIARP), had been first discovered as a prostate-specific cell surface antigen by suppressing subtractive hybridizations (Chen et al., 2014). Accumulating evidence has indicated that STEAP4 is a novel anti-obesity gene that is regulated by multiple factors, including nutritional stress, hormones, pro-inflammatory factors, and adipokines (Chen et al., 2010; Fasshauer et al., 2003; Kralisch et al., 2009; Moldes et al., 2001; Ramadoss et al., 2010). Moreover, STEAP4 is significantly downregulated in adipose tissue of obese patients and is involved in tissue metabolic regulation, such as improving glucose uptake, decreasing inflammatory response, and increasing insulin sensitivity (Chen et al., 2014). A previous study demonstrated that STEAP4 plays an important role in regulating the proliferation, apoptosis, and insulin sensitivity (Qin et al., 2010), but whether STEAP4 involves in the function of MDM2 in adipocytes is also unknown.

In our study, we constructed *Mdm2* adipocyte-specific knock-in (*Mdm2*-AKI) mice and found that *Mdm2*-AKI mice displayed increased body weight, decreased energy expenditure, and exacerbated insulin resistance both on a normal chow diet (NCD) and a HFD. The weights of WAT were increased and the size of adipocytes was hypertrophic in WAT in *Mdm2*-AKI mice on a NCD. Although the iWAT weight was augmented, the eWAT weight was decreased and indistinguishable in *Mdm2*-AKI mice on a HFD for 12 weeks and 8 months, respectively. Furthermore, under HFD feeding condition, MDM2 overexpression induced eWAT dysfunction such as cellular senescence, apoptosis, and inflammation, through ubiquitin-mediated STEAP4 degradation, eventually, exacerbating hepatic steatosis and insulin resistance.

## Results

### Adipose murine double minute 2 expression is related to nutritional status change and adipocyte differentiation

**Figure 1:**
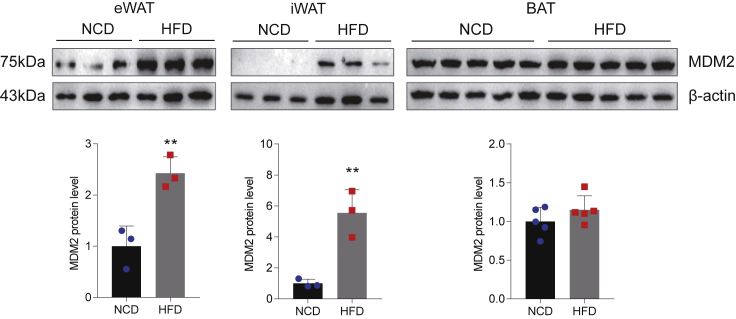
Adipose MDM2 expression is related to a nutritional status change The protein expression levels of MDM2 in adipose tissues of mice on a NCD and on a HFD were detected by Western blotting. n = 3. Data are represented as mean ± SD. Statistical analysis was carried out by Student’s *t* test. ∗∗p < 0.01.

To investigate the role of MDM2 in adipose tissue function, we first examined the expression of MDM2 in mouse adipose tissue induced by HFD. Similar to a recent study (Hallenborg et al., 2021), HFD increased the expression of MDM2 in WAT, including eWAT and iWAT, not in BAT, compared to NCD (Figure 1). Previous studies have suggested that MDM2 is involved in the initiation of adipocyte differentiation (Hallenborg et al., 2012, 2016). Similar to peroxisome proliferator-activated receptor γ (PPARγ), an adipogenic marker, the expression of MDM2 was also increased during adipocytes differentiation (Figure S1). The above results indicated that MDM2 had a crucial role in adipose tissue function.

### Adipose-specific murine double minute 2 transgenic mice display decreased energy expenditure and exacerbated insulin resistance

**Figure 2:**
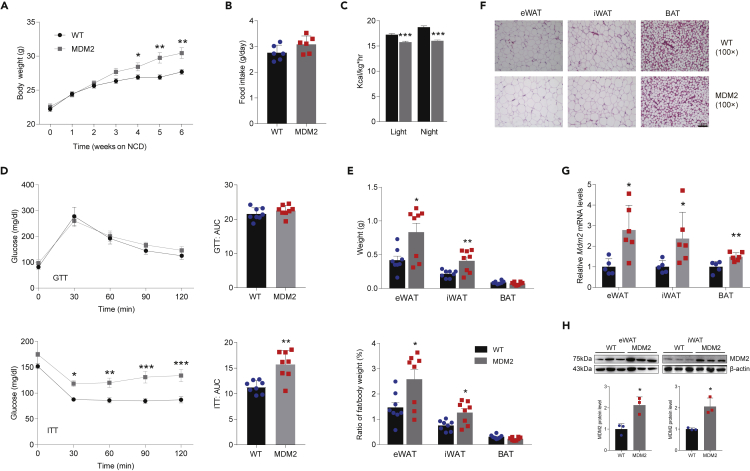
Adipose-specific MDM2 overexpression aggravates HFD-induced energy expenditure decrease and insulin resistance (A) Body weight of WT mice and *Mdm2*-AKI mice on a NCD. n = 8/group. Data are represented as mean ± SD. Statistical analysis was carried out by two-way ANOVA. ∗p < 0.05 and ∗∗p < 0.01. (B) Food intake of WT and *Mdm2*-AKI mice on a NCD. n = 6/group. Data are represented as mean ± SD. Statistical analysis was carried out by Student’s *t* test. (C) Heat production of WT and *Mdm2*-AKI mice on a NCD was measured by CLAMS. n = 8/group. Data are represented as mean ± SEM. Statistical analysis was carried out by Student’s *t* test. ∗∗∗p < 0.001. (D) GTT (up), ITT (down), and area under the curve (AUC) of WT and *Mdm2*-AKI mice on a NCD were analyzed. n = 8/group. Data are represented as mean ± SD. Statistical analysis was carried out by two-way ANOVA for GTT and ITT, and Student’s *t* test for AUC. ∗p < 0.05, ∗∗p < 0.01 and ∗∗∗p < 0.001. (E) Weight (up) and ratio (down) of fat weight/body weight of WAT in WT and *Mdm2-*AKI mice on a NCD. n = 8/group. Data are represented as mean ± SD. Statistical analysis was carried out by one-way ANOVA. ∗p < 0.05 and ∗∗p < 0.01. (F) H&E staining of WAT of WT and *Mdm2*-AKI mice on a NCD. Scare bars, 100 μm. (G) The mRNA expression level of MDM2 in adipose tissues of WT and *Mdm2*-AKI mice on a NCD. n = 6/group. Data are represented as mean ± SEM. Statistical analysis was carried out by Student’s *t* test. ∗p < 0.05 and ∗∗p < 0.01. (H) The protein expression level of MDM2 in adipose tissues of WT and *Mdm2*-AKI mice on a NCD. n = 3/group. Data are represented as mean ± SD. Statistical analysis was carried out by Student’s *t* test. ∗p < 0.05.

To further determine the function of MDM2 in adipose tissue, *Mdm2*-AKI mice were created under the control of adiponectin promoter and the genotype of *Mdm2*-AKI mice was identified using PCR (Figures S2A and S2B). Next, we firstly investigated the functional significance of adipocyte-specific MDM2 rise for energy homeostasis in a NCD status. Compared to WT mice, *Mdm2*-AKI mice fed on NCD displayed an increased tendency in body weight, not in food intake (Figures 2A and 2B). *Mdm2*-AKI mice had significantly lower heat production (Figure 2C). Additionally, glucose tolerance tests (GTTs) showed no difference between *Mdm2*-AKI mice and WT mice, and insulin tolerance tests (ITTs) indicated that insulin sensitivity was lower in *Mdm2*-AKI mice than in WT mice (Figure 2D). Furthermore, we detected the adipocyte architecture of *Mdm2*-AKI mice. After 8 weeks of NCD, *Mdm2*-AKI mice had greater WAT weight and WAT/body weight ratios compared to WT mice (Figure 2E), which indicated that weight gain was owing to the increase in WATs weight. Meanwhile, H&E staining showed that adipocytes were obviously and slightly hypertrophic in WAT and BAT of *Mdm2*-AKI mice on a NCD, respectively (Figure 2F). Additionally, the mRNA and protein expression levels of MDM2 were increased in adipose tissue of *Mdm2*-AKI mice on a NCD (Figures 2G and 2H).

### Adipose-specific murine double minute 2 overexpression aggravates high-fat diet-induced energy expenditure decrease and insulin resistance

**Figure 3:**
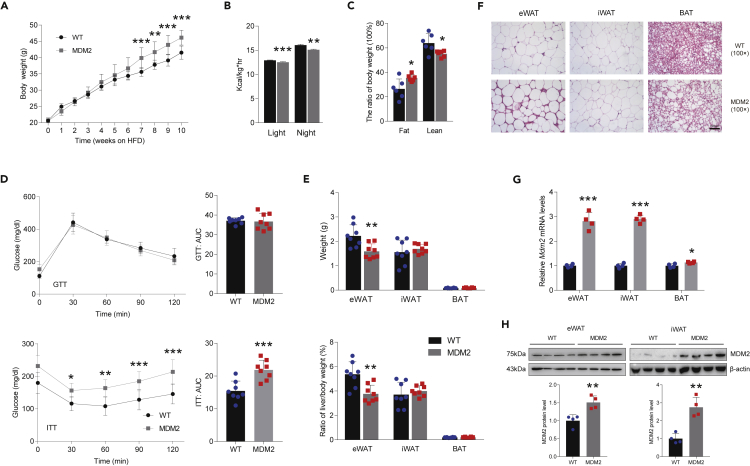
Adipose-specific MDM2 overexpression aggravates HFD-induced energy expenditure decrease and insulin resistance (A) Body weight of WAT in WT and *Mdm2*-AKI mice on a HFD for 10 weeks. n = 8/group. Data are represented as mean ± SD. Statistical analysis was carried out by two-way ANOVA. ∗∗p < 0.01 and ∗∗∗p < 0.001. (B) Heat generation of WT and *Mdm2*-AKI mice on a HFD for 12 weeks were measured by CLAMS. n = 8/group. Data are represented as mean ± SEM. Statistical analysis was carried out by Student’s *t* test. ∗∗p < 0.01 and ∗∗∗p < 0.001. (C) Fat and lean mass of WT and *Mdm2*-AKI mice on a HFD for 12 weeks were determined by noninvasive EchoMRI. n = 6/group. Data are represented as mean ± SD. Statistical analysis was carried out by Student’s *t* test. ∗p < 0.05. (D) GTT (up), ITT (down) and AUC of WT and *Mdm2*-AKI mice on a HFD were analyzed. n = 8/group. Data are represented as mean ± SD. Statistical analysis was carried out by two-way ANOVA for GTT and ITT, and Student’s *t* test for AUC. ∗p < 0.05, ∗∗p < 0.01 and ∗∗∗p < 0.001. (E) Weight (up) and ratio (down) of fat weight/body weight of WAT in WT and *Mdm2*-AKI mice on a HFD for 12 weeks. n = 8/group. Data are represented as mean ± SD. Statistical analysis was carried out by one-way ANOVA. ∗∗p < 0.01. (F) H&E staining of WAT of WT and *Mdm2*-AKI mice on a HFD for 12 weeks. Scare bars, 100 μm. (G) The mRNA expression level of MDM2 in adipose tissues of WT and *Mdm2*-AKI mice on a HFD. n = 4/group. Data are represented as mean ± SEM. Statistical analysis was carried out by Student’s *t* test. ∗p < 0.05 and ∗∗∗p < 0.001. (H) The protein expression level of MDM2 in adipose tissues of WT and *Mdm2*-AKI mice on a HFD. n = 4/group. Data are represented as mean ± SD. Statistical analysis was carried out by Student’s *t* test. ∗∗p < 0.01.

Next, we tested the effect of HFD on *Mdm2*-AKI mice. Like *Mdm2*-AKI mice fed a NCD, *Mdm2*-AKI mice on a HFD for 12 weeks or 8 months exhibited greater body weight and lower heat production, and had more fat mass and less lean mass (Figures 3A–3C and S3A–S3C). And *Mdm2*-AKI mice on a HFD for both 12 weeks and 8 months displayed lower insulin sensitivity and had no difference in glucose tolerance (Figures 3D and S3D). Moreover, both weight and tissue/body weight ratio of iWAT between *Mdm2*-AKI and WT mice were increased in a HFD for 12 weeks and 8 months (Figures 3E and S3E), which were consistent with the body weight increase. On the other hand, the eWAT weight was decreased and indistinguishable in *Mdm2*-AKI mice on a HFD for 12 weeks and 8 months, respectively (Figures 3E and S3E). And the eWAT weight/body weight ratio was lower in *Mdm2*-AKI mice on a HFD (Figures 3E and S3E). Meanwhile, no difference in the weight of BAT was noted between *Mdm2*-AKI and WT mice on a HFD for 12 weeks and 8 months, and there was a lower tissue/body weight ratio of BAT in *Mdm2*-AKI on a HFD for 8 months (Figures 3E and S3E). H&E staining showed that the size of WAT adipocytes was similar in WT and *Mdm2*-AKI mice on a HFD (Figures 3F and S3F), while the size of BAT adipocytes was increased in *Mdm2*-AKI mice on a HFD (Figures 3F and S3F). The increased expression levels in mRNA and protein of MDM2 were also confirmed in adipose tissue of *Mdm2*-AKI mice on a HFD (Figures 3G and 3H).

### Adipose-specific murine double minute 2 overexpression aggravates high-fat diet-induced epididymal white adipose tissue senescence

**Figure 4:**
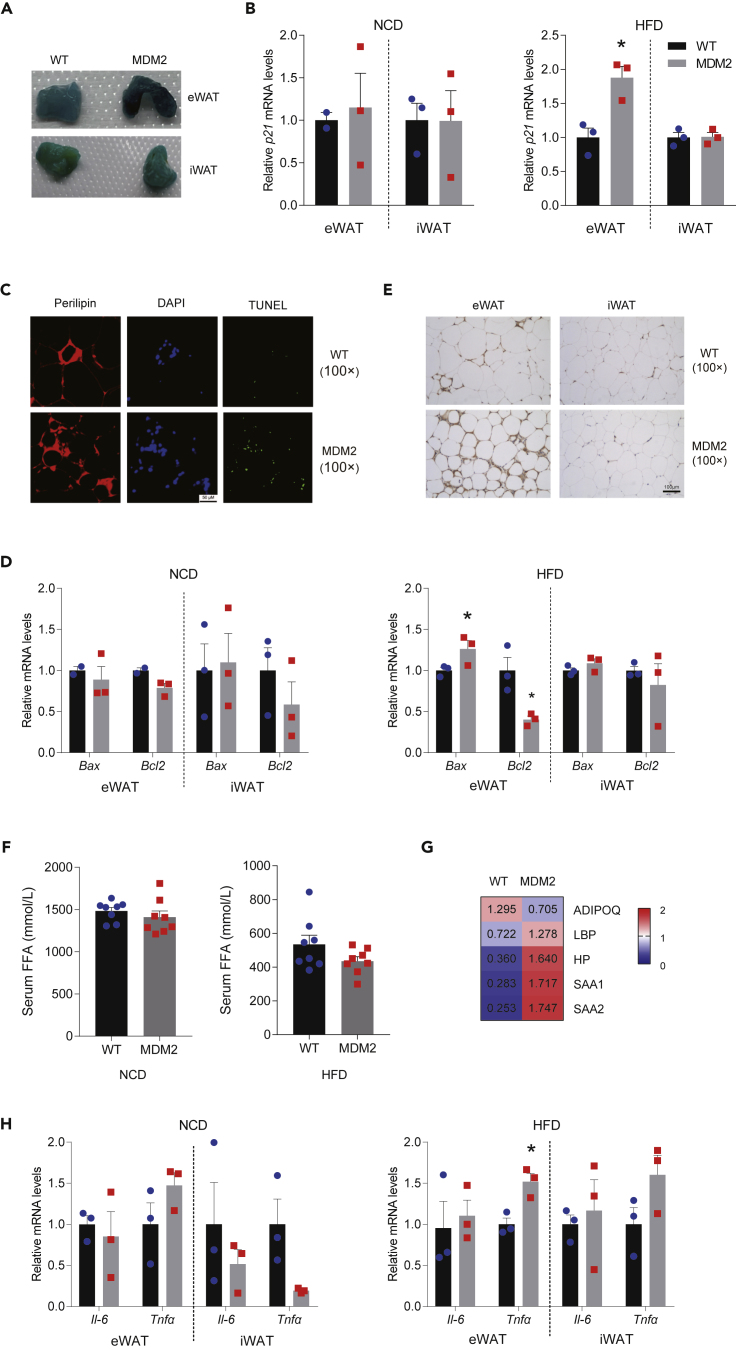
MDM2 overexpression in adipose tissues increases HFD-induced eWAT dysfunction (A) Senescence β-galactosidase staining in WAT of WT and *Mdm2*-AKI mice on a HFD for 12 weeks. (B) Relative mRNA levels of *p21* in WAT of WT and *Mdm2*-AKI mice on a NCD for 8 weeks and on a HFD for 12 weeks, relative to β-actin. n = 2 for WT on NCD, n = 3 for the others. Data are represented as mean ± SEM. Statistical analysis was carried out by Student’s *t* test. ∗p < 0.05. (C) Representative immunofluorescence staining of TUNEL (green) and perilipin (red) in eWAT of WT and *Mdm2*-AKI mice on a HFD for 12 weeks. Scare bars, 50 μm. (D) Relative mRNA levels of *Bax* and *Bcl2* in WAT of WT and *Mdm2*-AKI mice on a NCD for 8 weeks and on a HFD for 12 weeks, relative to β-actin. n = 2 for WT on NCD, n = 3 for the others. Data are represented as mean ± SEM. Statistical analysis was carried out by one-way ANOVA. ∗p < 0.05. (E) F4/80 antigen positivity in WAT of WT and *Mdm2-*AKI mice on a HFD for 12 weeks. Scare bars, 100 μm. (F) Biochemical analysis of serum FFA in WT and *Mdm2*-AKI mice on a NCD for 8 weeks and on a HFD for 12 weeks. n = 8/group. Data are represented as mean ± SD. Statistical analysis was carried out by Student’s *t* test. (G) Proteins were identified by LC-MS/MS in the serum of *Mdm2*-AKI mice on a HFD for 12 weeks. (H) Relative mRNA levels of *Tnfα* and *Il-6* in WAT of WT and *Mdm2*-AKI mice on a NCD for 8 weeks and on a HFD for 12 weeks, relative to β-actin. n = 3/group. Data are represented as mean ± SEM Statistical analysis was carried out by one-way ANOVA. ∗p < 0.05.

A previous study had shown that adipocyte-specific MDM2 deficiency triggers a series of aging-associated metabolic dysfunction (Liu et al., 2018). Here, we also investigated senescence and apoptosis of WAT in *Mdm2*-AKI mice. The results showed that more senescent cells were identified via β-galactosidase staining (Figures 4A and S4A) and the mRNA level of senescence-related gene cyclin-dependent kinase inhibitor 1A (*CDKN1A*, *p21*) was upregulated in the eWAT, but not iWAT, of *Mdm2*-AKI mice on a HFD (Figures 4B and S4B). And there was no change in the mRNA level of *p21* in eWAT and iWAT of WT and *Mdm2*-AKI mice on a NCD (Figure 4B). Furthermore, more apoptotic adipocytes were detected in the eWAT of *Mdm2*-AKI mice on a HFD, as revealed by the TUNEL assay (Figures 4C and S4C). Consistently, the mRNA level of the anti-apoptotic gene B-cell lymphoma-2 (*Bcl2)* decreased in the eWAT of *Mdm2*-AKI mice on a HFD for 12 weeks, whereas that of the pro-apoptotic gene BCL2 associated X *(Bax)* increased in the eWAT of *Mdm2*-AKI mice on a HFD for 12 or 8 months (Figures 4D and S4D).

Cellular senescence in adipose tissue has been known to cause failure in sequestering lipotoxic fatty acids, as well as inflammatory cytokine and chemokine generation (Tchkonia et al., 2010). We also tested the inflammation status of WAT of WT and *Mdm*2-AKI mice. The results showed that the eWAT, not iWAT, of *Mdm*2-AKI mice on a HFD for 12 weeks contained a markedly increased number of mononuclear cells among adipocytes (Figure 3F). The mononuclear cells were macrophages verified via immunohistochemistry using F4/80 (Figure 4E), suggesting macrophage infiltration in the eWAT of *Mdm2*-AKI mice. Moreover, there was no difference in macrophage infiltration between *Mdm2*-AKI and WT mice on a HFD for 8 months (Figure S4E).

Although there was no difference in serum-free fatty acid (FFA) level between *Mdm2*-AKI mice and WT controls (Figures 4F and S4F), the results from liquid chromatography-tandem mass spectrometry (LC-MS/MS) showed that adiponectin was downregulated and some pro-inflammatory factors, including haptoglobin, serum amyloid A protein 1/2 (SAA1/SAA2) and lipopolysaccharide-binding protein (LBP), were upregulated in the serum of the *Mdm2*-AKI mice on a HFD compared to WT controls (1.5-fold-change criterion, FDR <0.01) (Figure 4G). And qPCR demonstrated that HFD for 12 weeks induced an increase in *Tnfα*, not interleukin 6 (*Il-6*), mRNA expression level in eWAT of *Mdm2*-AKI mice (Figures 4H and S4G). Additionally, there was a decrease in the expression of PPARγ in eWAT, not iWAT, of *Mdm2*-AKI mice on a HFD, indicating that HFD, not NCD, impaired the differentiation and maturation of eWAT adipocytes (Figures S5A and S5B). Those differential effects in eWAT and iWAT underlined that MDM2 played a complex role in adipose tissue under HFD feeding condition.

### Adipocyte-specific murine double minute 2 overexpression aggravates hepatic steatosis

**Figure 5:**
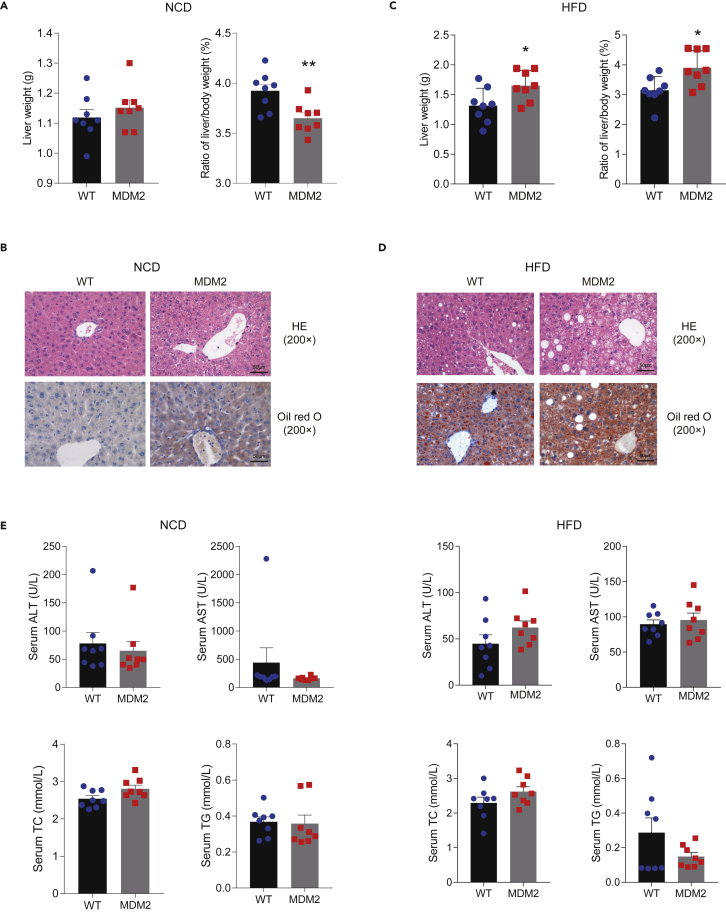
MDM2 overexpression in adipose tissues aggravates HFD-induced hepatic steatosis (A and C) Liver weight (left) and the ratio of liver weight/body weight (right) in WT and *Mdm2*-AKI mice on a NCD for 8 weeks (A) and on a HFD for 12 weeks (C). n = 8/group. Data are represented as mean ± SD. Statistical analysis was carried out by Student’s t test. ∗ p < 0.05 and ∗∗ p < 0.01. (B and D) H&E and oil red O staining of livers of WT and *Mdm2*-AKI mice on a NCD for 8 weeks (B) and HFD for 12 weeks (D). Scare bars, 50 μm. (E) Biochemical analysis of serum in WT and *Mdm2*-AKI mice on a NCD for 8 weeks and on a HFD for 12 weeks. n = 8/group. Data are represented as mean ± SD. Statistical analysis was carried out by Student’s *t* test. (ALT, Alanine aminotransferase; AST, Aspartate transaminase; TC, Total cholesterol; TG, Triglyceride).

Some studies have been identified related to cross-talk between adipose tissue and liver (Smith and Kahn, 2016), we investigated metabolic changes in the liver. Our results demonstrated that under NCD for 8 weeks, no difference in liver weight was observed between *Mdm2*-AKI and WT mice, while the liver/body weight ratio was lower in *Mdm2*-AKI mice (Figure 5A). Although H&E staining showed there was no obvious difference in liver between *Mdm2*-AKI mice on a NCD and WT mice on NCD, *Mdm2*-AKI mice on a NCD showed more lipid droplets in their liver compared to WT mice on a NCD determined through oil red O staining (Figure 5B). However, *Mdm2*-AKI mice had higher liver weight under a HFD for 12 and 8 months and a greater liver/body weight ratio under a HFD for 12 weeks (Figures 5C and S6A). Chronic HFD exposure in *Mdm2*-AKI mice apparently induced severe hepatic steatosis, including massive accumulations of large lipid droplets and increasing the ballooning degeneration of liver cells (Figures 5D and S6B). Biochemical analysis indicated that HFD for 8 months induced an increase in ALT and total cholesterol (TC) in *Mdm2*-AKI mice, compared to WT mice (Figures 5E and S6C). The results indicated the importance of adipose-liver organ crosstalk in maintaining systemic metabolic health.

### Quantitative proteomic profiling of murine double minute 2 regulated proteins in adipocyte tissue

**Figure 6:**
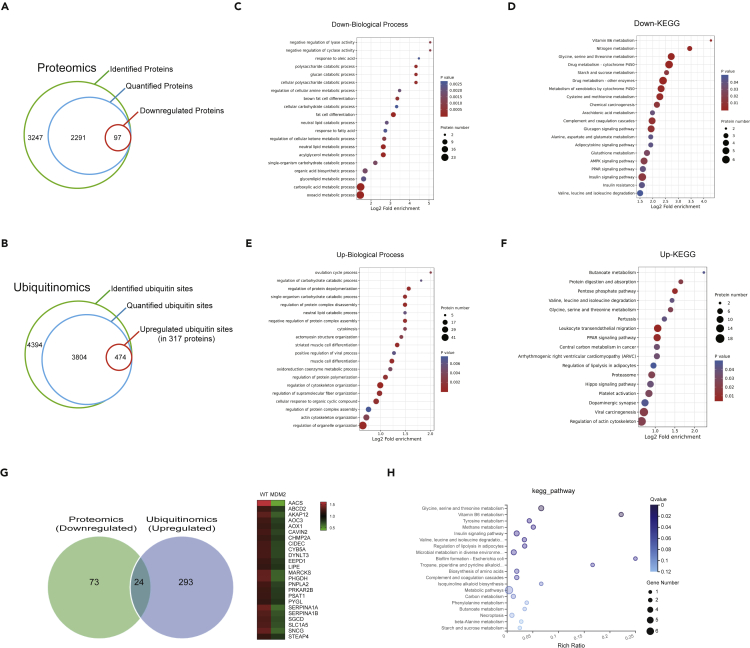
Functional annotation of altered proteome and ubiquitinome in eWAT of *Mdm2*-AKI mice (A) Number of identified proteins, quantified proteins, and downregulated proteins. (B) Number of identified ubiquitin sites, quantified ubiquitin sites, and upregulated ubiquitin sites. (C) GO analysis of the significantly downregulated proteins for biological process. (D) KEGG analysis of the significantly downregulated proteins. (E) GO analysis of the proteins, which had the significantly upregulated ubiquitin site modification, for biological process. (F) KEGG analysis of the proteins, which had the significantly upregulated ubiquitin site modification. (G) Venn diagram of downregulated proteins identified via proteomics and proteins with increased ubiquitination modification identified via ubiquitinomics in eWAT of WT and *Mdm2*-AKI mice on a HFD for 12 weeks. (H) KEGG analysis of the 24 modification proteins with downregulated expression and increased ubiquitination identified via proteomics and ubiquitinomics.

It is well known that MDM2, a known E3 ubiquitin ligase, can degrade substrates through interacting with substrates and ubiquitinating substrates. To develop the molecular mechanism of MDM2-induced eWAT dysfunction, we performed quantitative proteomics analysis in eWAT of *Mdm2*-AKI and control mice on HFD for 12 weeks through LC-MS/MS to system-wide identify the degradation substrates of MDM2. In this study, we identified 3247 proteins, and 2291 proteins were quantifiable (Figure 6A). With a criterion of ≥1.5-fold change (FDR <0.01), 97 downregulated proteins and 110 upregulated proteins in the group were identified (Figure 6A).

### Characterization of ubiquitinome in response to murine double minute 2 overexpression

To further characterize the deubiquitination substrates of MDM2, we employed an affinity-based ubiquitinated peptide enrichment approach to systematically quantify the change of ubiquitinome in the eWAT of *Mdm2*-AKI and control mice on HFD for 12 weeks. Here, we identified 4394 ubiquitination sites, and 3804 sites were quantifiable (Figure 6B). Subsequently, we obtained 474 significant upregulated ubiquitinated sites, in 317 proteins (1.5-fold-change criterion, FDR <0.01) in eWAT of *Mdm2*-AKI mice compared to WT mice (Figure 6B), which was likely to be modificated by MDM2.

### Annotation of murine double minute 2 regulated proteome and ubiquitinome

Next, we subjected the down-regulated proteins between WT and *Mdm2*-AKI groups to bioinformatics enrichment analysis with gene ontology (GO) and Kyoto Encyclopedia of Genes and Genomes (KEGG) databases. The results showed that these down-regulated proteins in eWAT were involved in multiple biological processes, such as fat cell differentiation, response to fatty acid, and neutral lipid metabolic process, in GO analysis (Figure 6C) and multiple signaling pathways, such as adipocytokine, adenosine 5′-monophosphate (AMP)-activated protein kinase (AMPK), peroxisome proliferator-activated receptor (PPAR), insulin and insulin resistance, in KEGG analysis (Figure 6D). And then, bioinformatics analysis of upregulated ubiquitinated proteins in response to MDM2 showed that they were closely related to the processes of neutral lipid catabolic process, cytokinesis, oxidoreduction coenzyme metabolic process, and muscle cell differentiation in GO analysis (Figure 6E) and involved in the signaling pathway of PPAR and regulation of lipolysis in adipocytes in KEGG analysis (Figure 6F).

Combining the results of proteomics and ubiquitinomics, we identified 24 down-regulated proteins, whose ubiquitin modification was increased in the *Mdm2*-AKI group (Figure 6G and Table S1). Moreover, KEGG analysis showed that these proteins were involved in the regulation of adipocyte lipolysis and insulin signaling pathway (Figure 6H). Together, the proteome and ubiquitinome analysis also suggested MDM2 had important roles in adipose tissue physiology.

### Identification of six-transmembrane epithelial antigen of prostate 4 as a murine double minute 2 target

**Figure 7:**
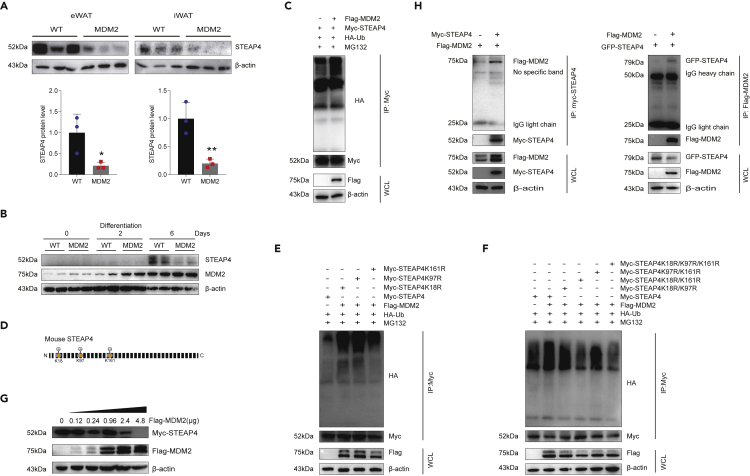
MDM2 promotes ubiquitin-mediated STEAP4 degradation (A) MDM2 overexpression decreased SPEAP4 protein levels in WAT. n = 3/group. Data are represented as mean ± SD. Statistical analysis was carried out by Student’s *t* test. ∗p < 0.05 and ∗∗p < 0.01. (B) MDM2 overexpression inhibited STEAP4 expression in differentiated adipocytes. (C) MDM2 overexpression promoted STEAP4 ubiquitination in 293T cells. (D) Sketch map showing the MDM2-catalyzed ubiquitinated sites on STEAP4 predicted using ubiquitinomics. (E and F) Detailed ubiquitination sites of STEAP4 modified by MDM2. (G) Inhibitory effects of MDM2 on STEAP4 protein levels were dose-dependent. (H) Interaction between MDM2 and STEAP4 was confirmed through co-immunoprecipitation.

Previous studies had indicated that STEAP4 has a crucial role in adipose tissue metabolism, we reasoned STEAP4 as a major MDM2 substrate candidate for further investigation. Consistent with the results of proteomics, the protein expression levels of STEAP4 were reduced in eWAT and iWAT of *Mdm2*-AKI mice fed on HFD (Figure 7A). Additionally, the expression of MDM2 and STEAP4 was increased in WT mature adipocytes compared to WT undifferentiated preadipocytes; however, MDM2 overexpression inhibited STEAP4 increase in differentiated 6 days (Figure 7B).

Furthermore, we evaluated the effects of MDM2 on the ubiquitin modification of STEAP4 in 293T cells. The results showed that MDM2 overexpression promoted ubiquitination of STEAP4 (Figure 7C). The results of ubiquitinomics indicated that the ubiquitination level of three sites, K18, K97, and K161, in STEAP4 were increased in *Mdm2*-AKI eWAT (Figure 7D). To confirm the detailed ubiquitination sites of STEAP4 modified by MDM2, different mutants (lysine (K) substituted by arginine (R)) for STEAP4 were constructed. Our results showed that the individual mutant of K18R, K97R, or K161R could still be ubiquitin modified by MDM2 overexpression (Figure 7E). When K18R and K161R co-existed, the ubiquitin modification of STEAP4 by MDM2 decreased, indicating that MDM2 mainly ubiquitinated STEAP4 in K18 and K161 sites (Figure 7F). The inhibitory effect of MDM2 on STEAP4 protein levels was dose-dependent (Figure 7G). Furthermore, co-immunoprecipitation showed that STEAP4 interacted with MDM2 in 293T cells treated with palmitic acid (PA) (Figure 7H).

### Six-transmembrane epithelial antigen of prostate 4 restoration in epididymal white adipose tissue alleviates murine double minute 2-aggravated adipose tissue dysfunction and hepatic steatosis

**Figure 8:**
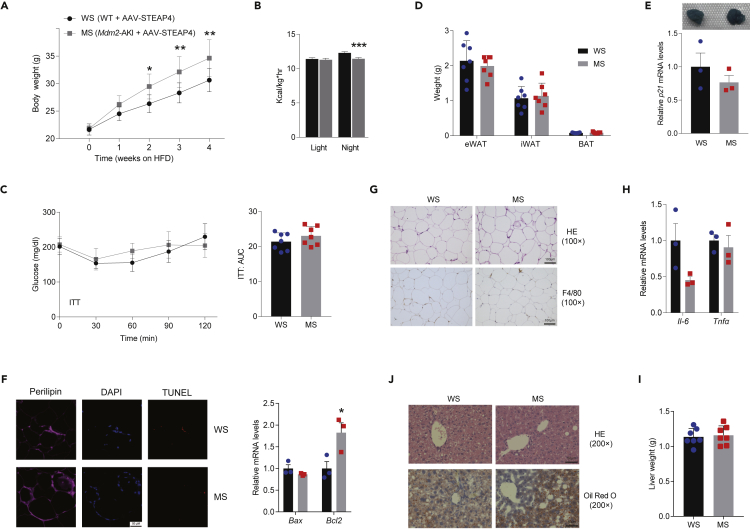
STEAP4 overexpression in eWAT alleviates MDM2-aggravated adipose tissue dysfunction and hepatic steatosis (A) Body weight of WT mice injected with AAV-STEAP4 (WS) and *Mdm2*-AKI mice injected with AAV-STEAP4 (MS). n = 7/group. Data are represented as mean ± SD. Statistical analysis was carried out by two-way ANOVA. ∗p < 0.05 and ∗∗p < 0.01. (B) Heat production of WS and MS mice measured by CLAMS. n = 7/group. Data are represented as mean ± SEM. Statistical analysis was carried out by Student’s *t* test. ∗∗∗p < 0.001. (C) ITT and AUC of WS and MS mice were analyzed. n = 7/group. Data are represented as mean ± SD. Statistical analysis was carried out by two-way ANOVA for GTT and ITT, and Student’s *t* test for AUC. (D) Weight of WAT in WS and MS mice. n = 7/group. Data are represented as mean ± SD. Statistical analysis was carried out by one-way ANOVA. (E) Senescence β-galactosidase staining and relative mRNA level of *p21* in eWAT of WS and MS mice. n = 3/group. Data are represented as mean ± SEM. Statistical analysis was carried out by Student’s t test. (F) Representative immunofluorescence staining (Left) of TUNEL (Red) and perilipin (pink), and relative mRNA levels of *Bax* and *Bcl2* (right) in eWAT of WS and MS mice, relative to β-actin. n = 3/group. Data are represented as mean ± SEM. Statistical analysis was carried out by Student’s *t* test. ∗p < 0.05. Scare bars, 50 μm. (G) H&E staining and F4/80 antigen immunohistochemistry in eWAT of WS and MS mice. Scare bars, 100 μm. (H) Relative mRNA levels of *Tnfα* and *Il-6* in eWAT of WS and MS mice, relative to β-actin. n = 3/group. Data are represented as mean ± SEM. Statistical analysis was carried out by Student’s *t* test. (I) Liver weight of WS and MS mice. n = 7/group. Data are represented as mean ± SD. Statistical analysis was carried out by Student’s *t* test. (J) H&E and oil red O staining of liver of WS and MS mice. Scare bars, 50 μm.

To determine whether STEAP4 mediates the effects of MDM2-induced adipocyte dysfunction and insulin resistance, the limited rescue of STEAP4 expression of *Mdm2*-AKI mice was performed through site-directed injection of AAV9-STEAP4 in eWAT and subjected to HFD challenge for 12 weeks. By comparing WT mice injected with AAV9-STEAP4 (WS) with *Mdm2*-AKI mice injected by AAV9-STEAP4 (MS), we found that STEAP4 restoration in eWAT had no apparent effects on adipose-specific MDM2 overexpression-aggravated changes in body weight and heat production (Figures 8A and 8B), but alleviated MDM2-aggravated insulin intolerance (Figure 8C). Next, STEAP4 restoration abolished MDM2-induced decrease in eWAT weight and alleviated MDM2-induced senescence, apoptosis, and increase in macrophage infiltration in eWAT (Figures 8D–8G). Moreover, STEAP4 regaining abrogated MDM2-mediated increase in mRNAs of *Tnfα* and decrease in the protein of PPARγ (Figures 8H and S7). Meanwhile, there were no differences in the weight and lipid droplet accumulations of the liver between WS and MS mice (Figures 8I and 8J), indicating that STEAP4 restoration abolished MDM2-induced hepatic steatosis.

## Discussion

Here, our study suggested that *Mdm2*-AKI mice displayed increased body weight, decreased energy expenditure, and exacerbated insulin resistance both on a NCD and a HFD. And the weight was increased in WATs of *Mdm2*-AKI mice on a NCD and in both iWAT and BAT of *Mdm2*-AKI mice on a HFD. Ectopic fat accumulation has also been identified as an important factor in determining individual risk for developing metabolic co-morbidities associated with obesity (Bluher, 2009). At the onset of obesity, iWAT expansion mainly depends on hypertrophy, whereas eWAT expands rapidly by hypertrophy and hyperplasia. It was more interesting that *Mdm2*-AKI mice on a HFD exhibited exacerbated eWAT dysfunction, such as increased cellular senescence, apoptosis, and inflammation, thereby aggravating HFD-induced hepatic steatosis and insulin resistance. Collective literature have indicated that eWAT and iWAT have intrinsically different metabolic functions, including the secretion of adipokines and inflammatory cytokines, lipolysis rates, and thermogenic potential (Ghaben and Scherer, 2019). Energy surplus induces fibrosis and low-grade chronic inflammation in eWAT, not iWAT (Nahmgoong et al., 2022). Visceral fat obesity has been more strongly associated with ectopic fat deposition, lipotoxicity, and metabolic disease compared to generalized obesity, especially in old age (Tchkonia et al., 2010). Additionally, macrophage accumulation in omental, not subcutaneous, adipose tissue is associated with aggravated steatosis and fibro-inflammation in insulin-resistant obese subjects independently of altered glycemic status (Tordjman et al., 2009). Meanwhile, another study also suggests that autophagy-related 7 knockout increased iWAT weight and decreases eWAT weight with HFD feeding (Sakane et al., 2021). Additionally, our results also demonstrated MDM2 overexpression induced BAT adipocyte hypertrophy, which contributed partly to a metabolic disorder such as decreased heat production and increased insulin resistance. As a major tissue of adaptive thermogenesis, BAT can significantly contribute to whole-body energy expenditure in mice. Mice lacking BAT are highly susceptible to obesity, whereas mice with increased BAT function are protected against HFD-induced harmful metabolic effects, including obesity and insulin resistance (Wang and Seale, 2016).

Cellular senescence is an arguably normal adaptive response to different stressors, including metabolic stress and high fatty acid levels. Collective literature showed that obesity may represent a state of accelerated aging and that adipose progenitor cells and mature adipocytes undergo senescence during obesity (Palmer et al., 2015; Tobler et al., 2010; Warboys et al., 2014). Excessive calorie intake promotes DNA damage and the resulting cellular senescence in adipose tissue in mice (Chen et al., 2015; Palmer et al., 2015). Lipopolysaccharide increase induced by obesity can lead to cellular senescence and impaired lipid formation of stromal vascular fraction cells (Zhao and Chen, 2015). Therefore, cellular senescence of adipocytes or adipose progenitor cells may have a significant impact on adipose tissue balance and homeostasis. During aging and obesity, cellular senescence could also increase the production of SASP including inflammatory cytokines, extracellular matrix-modifying proteases, and reactive oxygen species, which promotes cell death around senescent cells, tissue remodeling, and activation of adaptive and innate immune responses that could spread cellular senescence locally and systemically (Tchkonia et al., 2010). Meanwhile, secreted SASP is thought to be a means by which senescent cells request clearance from the immune system. However, apoptosis-resistant senescent cells persist and accumulate in aging tissues (Schafer et al., 2017). Studies have recognized that metabolic diseases, particularly obesity and diabetes, promote strong inflammatory responses and insulin resistance (Gregor and Hotamisligil, 2011; Lontchi-Yimagou et al., 2013). The inflammatory response in adipocytes is essential for maintaining systemic metabolic homeostasis. Adipose tissue is an active and dynamic endocrine organ that can express and secrete several pro-inflammatory and anti-inflammatory factors, such as *Tnfα*, *Il-6*, and *adiponectin*, which allow adipose tissue to communicate with other organs [centrally (brain) and peripherally (such as liver and skeletal muscle)] (Ahima and Flier, 2000; Bluher, 2009; Guilherme et al., 2008). This causes hepatic steatosis given that the increased expression of inflammatory factors in adipose tissue may be reflected by increased plasma levels, thereby contributing to systemic effects. The current study showed that local elevated MDM2 expression decreased the expression of adiponectin and increased the expression of some pro-inflammatory factors, such as haptoglobin, SAA1, SAA2, and LBP, in serum. Among them, overexpression of adiponectin, the most abundant peptide secreted by adipocytes, protects against HFD-induced lipotoxic effects of lipid accumulation and improves insulin sensitivity in mice, primarily in the liver (Combs et al., 2004). Additionally, inflammatory cytokines could induce systemic effects, further impeding adipogenesis, and promote fat cell lipolysis, releasing fatty acids that aggravate fat tissue's pro-inflammatory state and cause systemic lipotoxicity (Tchkonia et al., 2010). Although MDM2 overexpression in adipose tissue affects insulin sensitivity, the hyperglycemic clamp test and plasma insulin levels had not been detected currently, thus whether MDM2 affects the function of islet β cell need to be further determined.

A previous study showed that adipocyte MDM2 completely deficiency-induced progressive lipodystrophy in adipose tissues, promoting multiple metabolic complications and multiorgan senescence, which were dependent on p53 (Liu et al., 2018). In this study, MDM2 overexpression decreased the protein level of p53 (Figure S8), and induced adipocyte hypertrophy both in iWAT and BAT. Although the eWAT weight was increased under the NCD feeding condition, the eWAT weight was decreased and unaltered in *Mdm2*-AKI mice on a HFD for 12 weeks and 8 months, respectively. However, *Mdm2*-AKI mice on a HFD exhibited exacerbated senescence in eWAT, indicating the complex role of MDM2 in adipocyte function by p53-dependent and -independent mechanisms. Previous studies manifested that MDM2 is involved in the initiation of adipocyte differentiation via a p53-independent mechanism (Hallenborg et al., 2012, 2016). Recent study demonstrated that adipose tissue-specific MDM2 haploinsufficiency (*Mdm2*^Adi+/−^) driven by Fabp4 promoter-Cre led to a marked increase in body weight, adipose tissue mass, glucose intolerance, and hepatic steatosis in young mice at least in part through promoting nuclear exclusion of the transcriptional cofactors, MORC2 and LIPIN1, and thereby possibly hampered adipocyte function by antagonizing LIPIN1-mediated PPARγ coactivation (Hallenborg et al., 2021). In addition, although the iWAT and BAT were enlarged in *Mdm2*^Adi+/−^ mice on a HFD, the size of the eWAT was indistinguishable from wild-type mice on a HFD. Here, adipocyte-specific MDM2 knock-in mice by adiponectin promoter displayed decreased energy expenditure and increased body weight, insulin resistance, and hepatic steatosis. Under HFD feeding condition, MDM2 overexpression caused eWAT dysfunction through ubiquitin-mediated degradation of STEAP4. Furthermore, STEAP4 restoration in eWAT of *Mdm2*-AKI mice improved MDM2-induced metabolic disorder, including adipocyte dysfunction, insulin resistance, and hepatic steatosis. Previous study had indicated that the downregulation of PPARγ coincides with the senescence induction (Shih et al., 2017). Our results showed that the expression levels of MDM2 and PPARγ were increased with the increase of the maturity of adipocytes differentiation. However, the expression of PPARγ was decreased in senescent eWAT of *Mdm2*-AKI mice on a HFD, which could be reversed by STEAP4 overexpression. The above complex results from MDM2 deficiency or increment may suggest the importance of MDM2 at the physiological level for maintaining adipose function.

STEAP4 is a critical modulator for coordinating inflammation and metabolism in adipose tissue, especially visceral adipose tissue (Chen et al., 2010; Moldes et al., 2001). Multitudinous factors, including inflammatory cytokines, hormones, or adipokines, can regulate STEAP4 expression (Chen et al., 2010; Kralisch et al., 2009; Moldes et al., 2001). Feeding can also induce STEAP4 expression, with such a physiologic response being lost in *ob/ob* mice or HFD-induced obesity mice (Wellen et al., 2007). Antagonizing STEAP4 function can reduce the translocation of GLUT4 and thus decrease insulin-stimulated glucose transport in adipocytes (Qin et al., 2010; Wellen et al., 2007). The lack of STEAP4 in adipocytes or visceral tissues promotes macrophage infiltration, augmented inflammation, increased inflammatory factor production, and impaired insulin sensitivity (Moreno-Navarrete et al., 2011; ten Freyhaus et al., 2012; Wellen et al., 2007). STEAP4 overexpression actively protects adipocytes against inflammatory challenges (Arner et al., 2008; Han et al., 2013; ten Freyhaus et al., 2012).

In summary, our results demonstrated that MDM2 had an important and complex role in adipose function. Adipocyte-specific MDM2 overexpression resulted in exacerbated weight gain, insulin resistance, and decreased energy expenditure. Furthermore, under HFD feeding condition, MDM2 overexpression caused eWAT dysfunction, such as cellular senescence, apoptosis, and inflammation through ubiquitin-mediated STEAP4 degradation, highlighting the crucial role of the MDM2-STEAP4 axis in maintaining healthy adipose tissue function and improving hepatic steatosis.

### Limitation of the study

There are many unknown details that need to be further developed, for example, how MMD2 regulates *Steap4* at the transcriptional level; how STEAP4 affects the senescence, apoptosis, and inflammation of adipose tissue; what causes the difference in functions of MDM2 between eWAT and iWAT.

## STAR★Methods

### Key resources table

**Table:** 

REAGENT or RESOURCE	SOURCE	IDENTIFIER
**Antibodies**		

anti-Perilipin A, dil: 1/1000	Abclonal	Cat#A4758; RRID: AB_2863342
anti-β-Actin, dil: 1/10000	Abclonal	Cat#AC026; RRID: AB_2768234
anti-F4/80, dil:1/500	Servicebio	Cat#GB11027; RRID: AB_2814687
anti-MDM2, dil:1/1000	Abcam	Cat#ab38618; RRID: AB_776258
anti-STEAP4, dil:1/1000	Thermo Fisher Scientific	Cat#PA5-106509; RRID:AB_2854178
anti-Tp53, dil:1/1000	Abclonal	Cat#A16989; RRID: AB_2772689
anti HA-tag, dil:1/1000	Abclonal	Cat#AE036; RRID: AB_2771924
anti-Flag, dil:1/1000	Abclonal	Cat#AE004; RRID: AB_2771921
anti-Myc-tag, dil:1/1000	Solarbio	Cat#K106458P; RRID: N/A
anti-GFP, dil:1/1000	Sigma-Aldrich	Cat# G1544; RRID: AB_439690
anti-PPARγ, dil:1/1000	Abclonal	Cat#A11183; RRID: AB_2758449

**Bacterial and virus strains**		

*Trans5α*	Transgen Biotech	Cat #CD201
AAV9-CMV-m*Steap4*-MYC	Hanbio Biotechnology (shanghai) Co.,LTD	N/A

**Chemicals, peptides, and recombinant proteins**		

Fetal Bovine Serum (FBS)	Gibco	Cat#10091148
Palmitic acid (PA)	Sigma-Aldrich	Cat# P5585
MG132	MCE	Cat#133407-82-6
Trizol	Invitrogen	Cat#15596018
PMSF	Beyotime Biotechnology	Cat#ST506
cComplete Tablets EDTA-free, EASYpack	Roche	Cat#4693132001
SYBR Green PCR Master Mix	Promega	Cat#A6002
5 x SDS-Loading Buffer	Beyotime Biotechnology	Cat#P0015
protein ladder	Thermo Fisher Scientific	Cat#26616
Insulin	Sigma-Aldrich	Cat#I-5500
RIPA buffer	Beyotime Biotechnology	Cat#P0013
Polybrene	Millipore	Cat#ISEQ00010
Direct PCR Lysis Reagent (Tail)	Viagen Biotech	Cat#102-T
DMEM/High Glucose	Gibco	Cat#C11995500BT
DMEM/F12(HAM) 1:1	Viva Cell	Cat#C3130
Cell lysis buffer for Western and IP	Beyotime Biotechnology	Cat#P0013
Collagenase I	Sigma-Aldrich	Cat#C0130
Pyruvic acid sodium salt	Solarbio	Cat#P8380
Dexamethasone	Sigma-Aldrich	Cat#D4902
Isobutylmethylxanthine	Sigma-Aldrich	Cat#I5879
Rosiglitazone	Sigma-Aldrich	Cat#R2408
3,3′,5-Triiodo-L-thyronine(T3)	Sigma-Aldrich	Cat#T2877
Immobilon ECL Ultra Western HRP Substrate	Millipore	Cat# WBULS0500

**Critical commercial assays**		

BCA protein assay kit	CWBIO	Cat#CW0014S
Seamless Assembly Cloning Kit	clone smarter	Cat#C5891
Senescence β-Galactosidase Staining Kit	Beyotime Biotechnology	Cat#C0602
High Capacity cDNA Reverse Transcription Kit	Applied Biosystems	Cat#4368813
TUNEL staining kit	Servicebio	Cat#G1504/G1502
TIANprep Mini Plasmid Kit	TIANGEN	Cat#DP103-03
Endofree Maxi Plasmid Kit	TIANGEN	Cat#DP117
HiPure Gel Pure DNA Mini Kit	Magen	Cat#D2111-02

**Deposited data**		

Serum proteomics of wild type mice and *Mdm2* adipocyte-specific knock-in mice fed high-fat diet	This paper	PRIDE Accession viewer: PXD034069
The eWAT proteomics of wild type mice and *Mdm2* adipocyte-specific knock-in mice fed high-fat diet	This paper	PRIDE Accession viewer: PXD034074
The eWAT ubiquitinome of wild type mice and *Mdm2* adipocyte-specific knock-in mice fed high-fat diet	This paper	iProX Accession viewer: PXD034127

**Experimental models: Cell lines**		

HEK293T	ATCC	Cat#CRL-3216
Mouse: Stromal-vascular fraction cells	This paper	N/A

**Experimental models: Organisms/strains**		

Adipocyte-specific knock-in *Mdm2* (*Mdm2*-AKI) mice	This paper	N/A
C57BL/6		

**Oligonucleotides**		

Primers of plasmids for Co-Immunoprecipitation and *In vitro* ubiquitination assay, see Table S2	This paper	N/A
Primers for qPCR, see Table S3	This paper	N/A

**Recombinant DNA**		

Plasmid: pCMV3-Flag-mMDM2	Sino Biological	Cat#MG50533-NF
Plasmid: pCMV3-myc-mSTEAP4	Sino Biological	Cat#MG5A0592-NM
Plasmid: pCMV3-myc-mSTEAP4(K18R)	This paper	N/A
Plasmid: pCMV3-myc-mSTEAP4(K97R)	This paper	N/A
Plasmid:pCMV3-myc-mSTEAP4(K161R)	This paper	N/A
Plasmid: pEGFP-C1-STEAP4	This paper	N/A

**Software and algorithms**		

SPSS statistics v17.0	IBM Corporation	https://www.ibm.com/cn-zh/products/spssstatistics
AlphaEaseFC	AlphaEaseFC software	

**Other**		

Mouse high fat diet	Researsh Diet	Cat#D12492
PVDF membranes	Millipore	Cat#IPVH00010
Anti-DDDDK-tag mAb Magnetic Agarose	MBL International	Cat#M185-10R
Anti-myc mAb Magnetic Agarose	Bimake	Cat#B26301

### Resource availability

#### Lead contact

Further information and requests for resources and reagents should be directed to the lead contact, Xiaojun Liu (xiaojunliu@ibms.pumc.edu.cn).

#### Materials availability

This study did not generate new unique reagents.

### Experimental model and subject details

#### Mice

Adipocyte-specific knock-in *Mdm2* (*Mdm2*-AKI) mice were created using the CRISPR-Cas9 system. The gRNA to Hipp11, the donor vector containing Adiponectin promoter-mouse *Mdm2* CDS-polyA cassette, and Cas9 mRNA were co-injected into fertilized mouse eggs to generate targeted knock-in offspring. The obtained mice were identified by PCR followed by sequence analysis, which were bred to wild-type (WT) mice to test germline transmission. All mice were housed (≤4/cage) in the SPF facility and maintained on a 12 h light-dark cycle and a regular unrestricted diet. Unless otherwise noted, 8-week-old male mice were used for all experiment. All mice were fed either a normal chow diet (9% fat; Lab Diet) or HFD (60% fat, Research Diets) and libitum with free access to water. Eight-week-old male WT and *Mdm2*-AKI mice were divided into two groups and injected with AAV9-STEAP4, respectively. Mice were weighed preoperatively and injected intraperitoneally with 0.3% phenobarbital sodium (0.1-0.2 mL/10g or 50 mg/kg). After successful anesthesia, the mice were supine and fixed on a small workbench in a sterile operating table. The skin was disinfected with 75% alcohol. A median abdominal incision was made with a length of about 0.7cm and each layer of the abdominal wall was cut successively. The eWAT was located and each side of eWAT was injected at 3 points with a sterile BD Insulin needle syringe, and each site was injected with a total volume of 5 μL (1.7 × 10ˆ9 v.g/μL) AAV9-STEAP4. After injection, each layer of the abdominal wall of mice was sutured intermittently. All the surgeries were performed under sodium pentobarbital anesthesia, and all efforts were made to minimize suffering. No mice were excluded from the analysis. All animal experiments were conducted under protocols approved by the Animal Research Committee of the Institute of Laboratory Animals, Institute of Basic Medical Sciences Chinese Academy of Medical Sciences & School of Basic Medicine Peking Union Medical College (ACUC-A01-2021-017).

#### Stromal-vascular fraction cells

Brown stromal-vascular fraction (BSVF) cells were isolated and induced as previously described (Kong et al., 2014). Briefly, BAT pad were dissected from newborn mice (postnatal day 1), minced, and then digested for 30 min at 37°C in isolation buffer [123 mM NaCl, 5 mM KCl, 1.3 mM CaCl_2_, 5 mM glucose, 100 mM HEPES, 4% BSA, 1.5 mg/mL Collagenase I (Sigma)]. Digested tissue was filtered through a 100 μm cell strainer to remove large pieces, and the flow-through was then centrifuged for 10 min at 1000g to pellet the BSVF cells. BSVF cells were resuspended in DMEM (C11995500BT, Gibco) containing 1% penicillin-streptomycin (Pen/Strep), and 20% FBS (10091148, Gibco), and then plated onto 10cm tissue culture dishes. For preadipocyte differentiation, cells grown to 100% confluence (Day 0) were exposed to differentiation cocktail containing 2 μg/mL dexamethasone (D4902, Sigma-Aldrich), 0.5 mg/mL insulin (I6634, Sigma-Aldrich), 0.5mM isobutylmethylxanthine (I5879, Sigma-Aldrich), 1nM T3 (T2877, Sigma-Aldrich), in DMEM with 10% FBS. Two days after induction, cells were maintained in DMEM containing 1μM rosiglitazone (R2408, Sigma- Aldrich), 0.02μM insulin, 1nM T3 and 10% FBS until ready for harvest (generally day 6–7 post differentiation). Isolation of iWAT SVF and differentiation of primary white preadipocytes were also done as described previously (Yao et al., 2017). Briefly, the WAT pads were dissected from 4–5 weeks old mice, and then digested for 30 min at 37°C in isolation buffer [PBS,1% BSA, 0.8 mg/mL Collagenase I]. Digested tissue was filtered through a 100 μm cell strainer to remove large pieces, and the flow-through was then centrifuged for 10 min at 1000 g to pellet the stromal-vascular fraction (SVF) cells. SVF cells were resuspended in DMEM/F12 (C3130, Viva Cell) containing 1% Pen/Strep, and 10% FBS, and then plated onto 10cm cell culture dishes. For preadipocyte differentiation, cells grown to 100% confluence (Day 0) were exposed to differentiation cocktail containing 1 μM dexamethasone, 5 μg/mL insulin, 0.5mM isobutylmethylxanthine, 1μM rosiglitazone in DMEM/F12 containing 10% FBS. Two days after induction, cells were maintained in media containing 5 μg/mL insulin in DMEM/F12, 1% Pen/Strep, and 10% FBS until ready for harvest (generally day 6–7 post differentiation).

#### Mouse calorimetry

Mice were housed individually in metabolic chambers of an Oxymax system (Columbus Instruments). The first readings were taken after a 24-h acclimation period. Heat production was determined and normalized to the body weight.

#### Mouse whole-body composition

EchoMRI-100 quantitative magnetic resonance whole-body composition analyzer (EchoMRI Body Composition Analyzer E26-231-M) was used to measure whole-body water, fat, and lean mass. Each value was normalized to body weight.

#### Glucose tolerance test and insulin tolerance test

For glucose tolerance tests (GTTs), mice fasted for 16 h and received an intraperitoneal injection of glucose (1 g/kg). For insulin tolerance tests (ITTs), mice fasted for 4 h and received an intraperitoneal injection of human insulin (0.75 IU/kg). Blood glucose concentrations were measured from tail blood at the indicated times including 0, 30, 60, 90, and 120 min, using a One-Touch Ultra® glucometer (LifeScan Inc., Milpitas, CA).

#### Histopathologic analysis

Liver and adipose tissue sections were fixed in 4% paraformaldehyde, then embedded in paraffin and stained with H&E to visualize the general morphological and structural characteristics of tissues or cell components and lesions. For F4/80 staining, samples were first paraffin-embedded and 5-micron slices mounted on slides. Samples were deparaffinized and immunoperoxidase staining conducted using anti-F4/80 primary antibody and DAB kit. Lipid droplet accumulation in the liver was visualized using Oil red O staining of frozen liver sections that were prepared in optimum cutting temperature (O.C.T.) compound. β-galactosidase staining in freshly collected adipose tissues was performed using a β-galactosidase staining kit (C0602, Beyotime Biotechnology, Shanghai, China).

#### TUNEL staining

Adipose tissue sections were subjected to antigen retrieval by boiling in 10 mmol/L sodium citrate buffer (pH 4.5), followed by blocking with PBS containing 10% FBS and 3% BSA for 1 h. Next, the sections were incubated with an anti-Perilipin A antibody (A16295, Ablonal, Wuhan, China) overnight at 4°C. The slides were incubated with an anti-rabbit IgG conjugated with Cy3 (GB21303, Servicebio, Wuhan, China) or Cy5 red fluorescent dye (GB27303, Servicebio) at room temperature for 1 h, followed by TUNEL staining kits (G1504/G1502, Servicebio) according to the manufacturer’s instructions.

#### Quantitative RT-PCR

Total RNA was extracted from mouse adipose using a Trizol-based method. Approximately 2 μg of total RNA was reverse-transcribed into a first-strand cDNA pool using reverse transcriptase and random primers, according to the manufacturer’s instructions. Q-PCR was performed using SYBR Green PCR Master Mix (A6002, Promega) with the gene-specific primers (Table S2). All gene expression data were normalized to actin expression levels.

#### Western blotting

Protein was extracted from frozen adipose samples in cell lysis buffer. In total, protein was loaded onto a 10% SDS-polyacrylamide gel, and separated proteins were transferred to PVDF membranes. Western blot assays were performed using specific antibodies. The proteins were quantified by AlphaEaseFC software.

#### Plasmid construction

Full-length sequences for mouse STEAP4 were inserted into the pEGFP-C1 vector using seamless assembly cloning. Variants of full-length STEAP4 with the conserved lysine residue changed to arginine were generated through standard PCR methods and were then subcloned into the pCMV3 vector using seamless assembly cloning. The primer sequences were seen in Table S3.

#### Cell culture

293T cells were cultured at 37°C, 5% CO_2_ in Dulbecco’s modified Eagle’s medium (DMEM) (Gibco, Carlsbad, USA) supplemented with 10% FBS, 1% penicillin-streptomycin. Subsequent to attachment, the cells were transfected with different plasmids as indicated.

#### Co-immunoprecipitation

293T cells were transfected with the indicated constructs using transfection reagent. Cells were treated with palmitic acid (PA, 0.4mM) for 24 h and then lysed with ice-cold lysis buffer, containing protease inhibitor cocktail (4693132001, Roche) and PMSF (ST506, Beyotime Biotechnology). Lysates were cleared by centrifugation, and protein was immunoprecipitated with the indicated Flag-tagged magnetic beads (M185-10R, MBL) or Myc-tagged magnetic beads (B26301, Bimake) at 4°C overnight. The beads were washed three times with pre-cooled PBS and then heated at 100°C in a loading buffer for 10min. Then, the beads were removed and the remaining immunocomplexes were collected and subjected to Western blotting with the indicated antibodies.

#### *In vitro* ubiquitination assays

293T cells were transfected with the indicated combinations of plasmids, including HA-ubiquitin, Flag-*Mdm2*, Myc-*Steap4* and Myc-*Steap4* mutants (K18R, K97R and K161R) plasmids. Cells were treated with 20 μM MG132 proteasome inhibitor (133407-82-6,MCE) for 6 h prior to lyse in lysis buffer (200 mM NaCl, 20 mM Tris-HCl (pH 7.4), 2.5 mM MgCl_2_, 0.5% Triton X-100, 1 mM PMSF, and protease inhibitor cocktail and then were sonicated. After centrifugation at 14,000 g, the cleared lysates were subjected to immunoprecipitation with anti-Myc mAb magnetic beads (B26301, Bimake). The beads were subsequently washed four times with lysis buffer. Then, the beads were removed and the remaining immunocomplexes were collected and subjected to Western blotting with the indicated antibodies.

#### Proteomics and ubiquitinome

The proteomics of mixed serum samples or mixed eWAT samples and the ubiquitinome of mixed eWAT samples were performed in Jingjie PTM BioLab (Hangzhou) Co. Ltd (www.ptm-biolab.com.cn). The difference was determined by 1.5-fold-change criterion, FDR <0.01.

### Quantification and statistical analysis

Data analyses were performed with SPSS (Version 17.0, SPSS, Inc.). The curves of body weight and tolerance tests were analysed using a repeated measure two-way ANOVA. For the other statistical analysis, the Kolmogorov-Smirnov test was firstly used for normality test. For the data conforming to the normal distribution, experiments were analyzed using Independent-Samples T test or one-way ANOVA. For the data not conforming to the normal distribution, experiments were analyzed using Mann-Whitney U test or Kruskal-Wallis test. All data presented were the mean ± SD except the mean ± SEM for mouse calorimetry and qPCR tests, and significance was represented with asterisks (∗) within the figures. p < 0.05 was considered statistically significant.

## Data Availability

Serum and the eWAT proteomics of wild type mice and *Mdm2* adipocyte-specific knock-in mice fed high-fat diet have been deposited to the ProteomeXchange Consortium (http://proteomecentral.proteomexchange.org) via PRIDE partner repository with the dataset identifier PXD034069 or PXD034074, respectively. The eWAT ubiquitinome of wild type mice and *Mdm2* adipocyte-specific knock-in mice fed high-fat diet has been deposited to the ProteomeXchange Consortium via the iProX partner repository with the dataset identifier PXD034127.
