# Supplementary material for: Murine double minute 2 aggravates adipose tissue dysfunction through ubiquitin-mediated six-transmembrane epithelial antigen of prostate 4 degradation
Source: iScience. 2022 Jun 7;25(7):104544. doi: 10.1016/j.isci.2022.104544 (PMC9209722; doi:10.1016/j.isci.2022.104544)
Supplement: Document S1. Figures S1–S8 and Tables S1–S3 [file mmc1.pdf]

iScience, Volume *25*

Supplemental information

Murine double minute 2 aggravates adipose tissue dysfunction through ubiquitin-mediated six-transmembrane epithelial antigen of prostate 4 degradation

Wei Zhao, Qiang Xu, Jiahui Yang, Xianghong Xie, Chunmei Li, Weihong Zhang, Enhui Chen, Yanfang Guo, Mingyue Gao, Jie Shi, Huabing Zhang, Hong Yao, Meixia Li, Li Yan, Fude Fang, Wenming Wu, and Xiaojun Liu

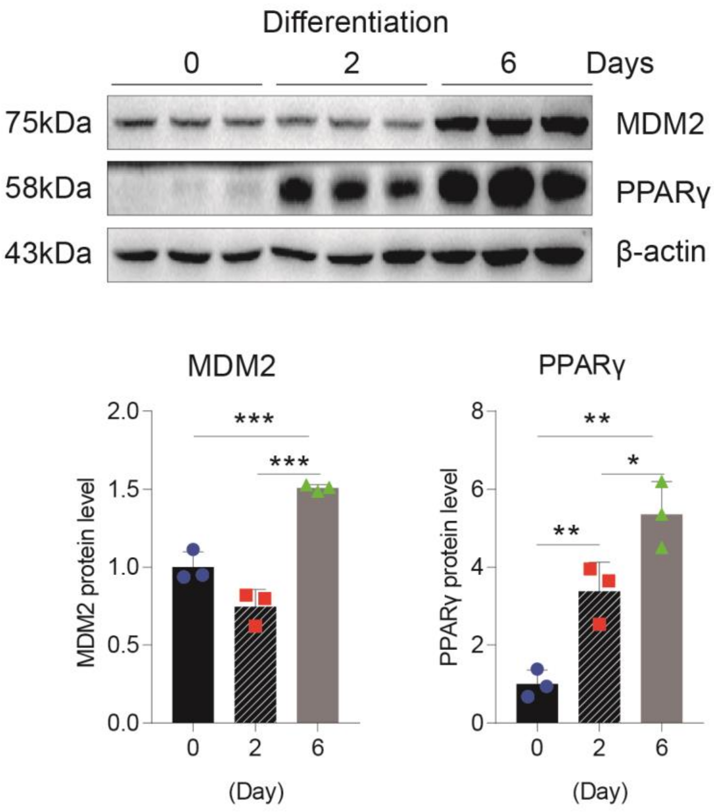

Figure S1. The MDM2 expression is increased with the maturity of adipocyte differentiation. Related to Figure 1.

Western blotting (up) and gray density (down) of MDM2 expression in SVF cells differentiation 0 day, 2 days and 6 days. n = 3. Data are represented as means ± SD. Statistical analysis was carried out by one-way ANOVA. * $p < 0.05$, ** $p < 0.01$ and *** $p < 0.001$.

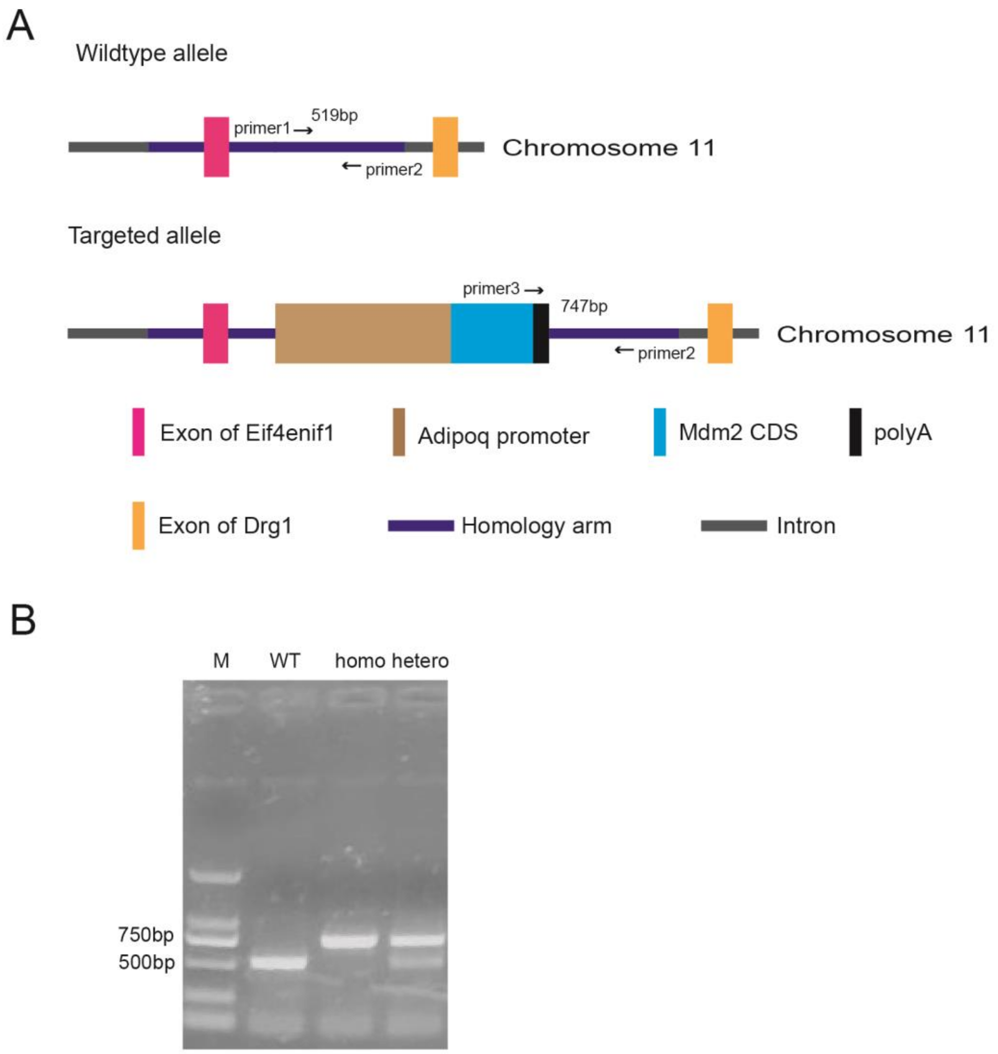

Figure S2. Creation of adipocyte-specific knock-in *Mdm2* mice. Related to Figure 2.

(A) Schematic diagram for the creation of adipocyte-specific knock-in *Mdm2* (*Mdm2*-AKI) mice.

(B) Identification of *Mdm2*-AKI mouse genotype.

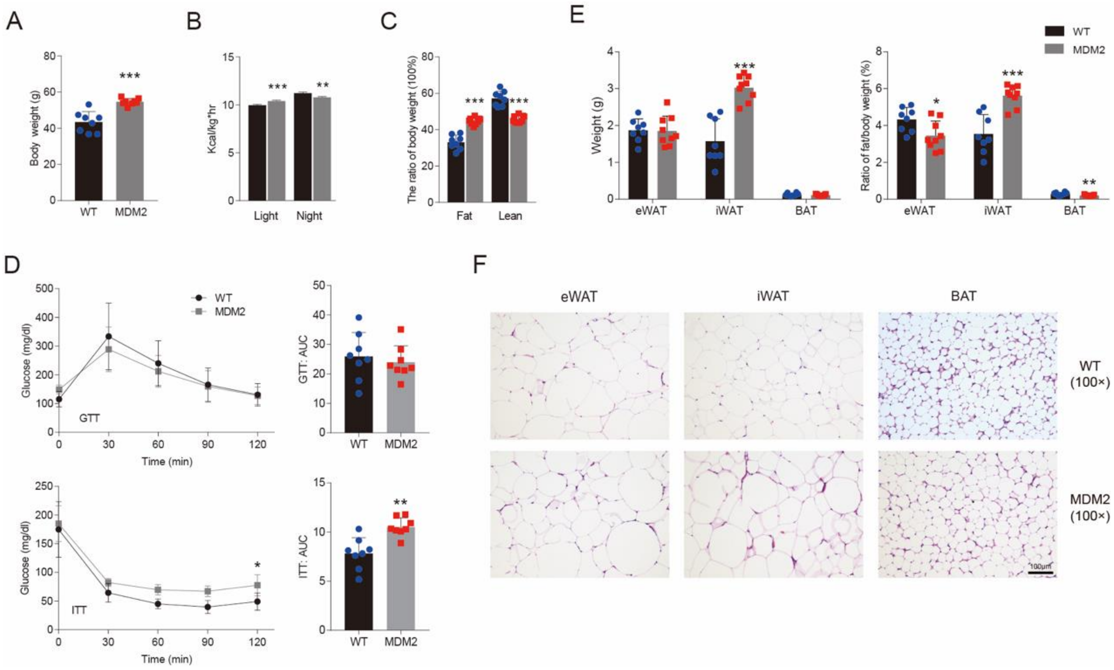

Figure S3. Adipose-specific MDM2 overexpression aggravates HFD-induced energy expenditure decrease and insulin resistance. Related to Figure 3.

(A) Body weight of WAT in WT and *Mdm2*-AKI mice on a HFD for 8 months (B). n = 8/group. Data are represented as mean ± SD. Statistical analysis was carried out by Student's t test. *** p < 0.001.

(B) Heat production of WT and *Mdm2*-AKI mice on a HFD for 8 months was measured by CLAMS. n = 8/group. Data are represented as mean ± SEM. Statistical analysis was carried out by Student's t test. ** p < 0.01 and *** p < 0.001.

(C) Fat and lean mass of WT and *Mdm2*-AKI mice on a HFD for 8 months were determined by noninvasive EchoMRI. n = 8/group. Data are represented as mean ± SD. Statistical analysis was carried out by Student's t test. *** p < 0.001.

(D) GTT (up), ITT (down) and area under the curve (AUC) of WT and *Mdm2*-AKI mice on a HFD for 8 months were analyzed. n = 8/group. Data are represented as mean ± SD. Statistical analysis was carried out by two-way ANOVA for GTT and ITT, and Student's t test for AUC. * p < 0.05 and ** p < 0.01.

(E) Weight (left) and ratio (right) of fat weight/body weight of WAT in WT and *Mdm2*-AKI mice on a HFD for 8 months. n = 8/group. Data are represented as mean ± SD. Statistical analysis was carried out by one-way ANOVA. * p < 0.05, ** p < 0.01 and *** p < 0.001.

(F) H&E staining of WAT and BAT of WT and *Mdm2*-AKI mice on a HFD for 8 months. Scare bars, 100 μm.

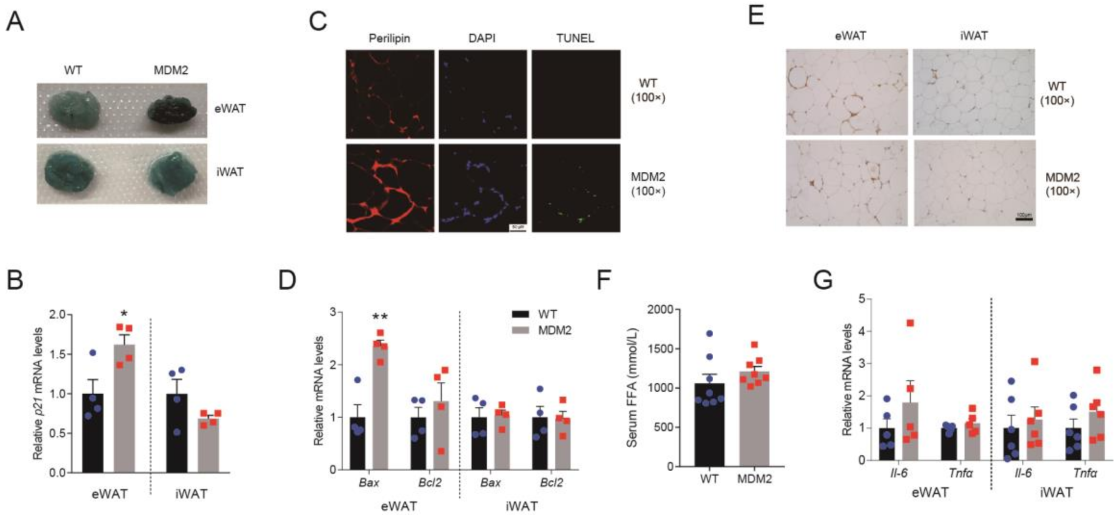

Figure S4. MDM2 overexpression in adipose tissues increases HFD-induced eWAT dysfunction. Related to Figure 4.

(A) Senescence β-galactosidase staining in WAT of WT and *Mdm2*-AKI mice on a HFD for 8 months.

(B) Relative mRNA levels of *p21* in WAT of WT and *Mdm2*-AKI mice on a HFD for 8 months, relative to β-actin. Data are represented as mean ± SEM. Statistical analysis was carried out by Student's t test. ** $p < 0.01$.

(C) Representative immunofluorescence staining of TUNEL (green) in eWAT of WT and *Mdm2*-AKI mice on a HFD for 8 months. Scare bars, 50 μm.

(D) Relative mRNA levels of *Bax* and *Bcl2* in WAT of WT and *Mdm2*-AKI mice on a HFD for 8 months, relative to β-actin. Data are represented as mean ± SEM. Statistical analysis was carried out by Student's t test. ** $p < 0.01$.

(E) F4/80 antigen positivity in WAT of WT and *Mdm2*-AKI mice on a HFD for 8 months. Scare bars, 100 μm.

(F) Biochemical analysis of serum FFA in WT and *Mdm2*-AKI mice on a HFD for 8 months. Data are represented as mean ± SD. Statistical analysis was carried out by Student's t test.

(G) Relative mRNA levels of *Tnfα* and *Il-6* in WAT of WT and *Mdm2*-AKI mice on a HFD for 8 months, relative to β-actin. Data are represented as mean ± SEM. Statistical analysis was carried out by Student's t test.

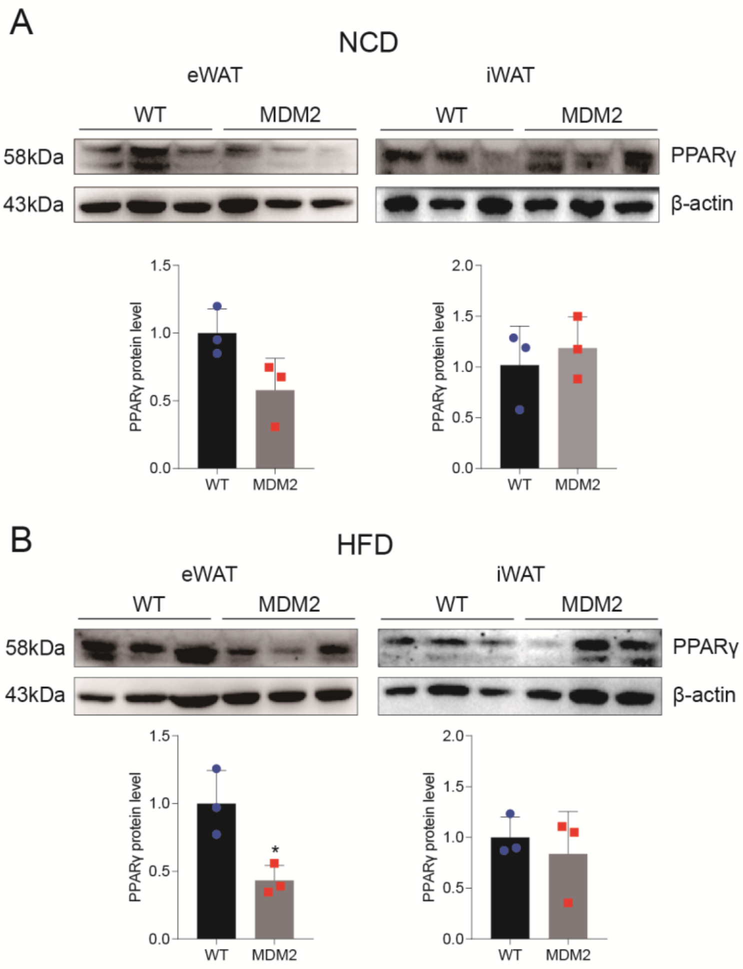

Figure S5. Western blotting (up) and protein gray value (down) of PPARγ in WAT of WT and *Mdm2*-AKI mice on a NCD for 8 weeks (A) and on a HFD for 12 weeks(B). Related to Figure 4.

n = 3/group. Data are represented as mean ± SD. Statistical analysis was carried out by Student's t test. * $p < 0.05$.

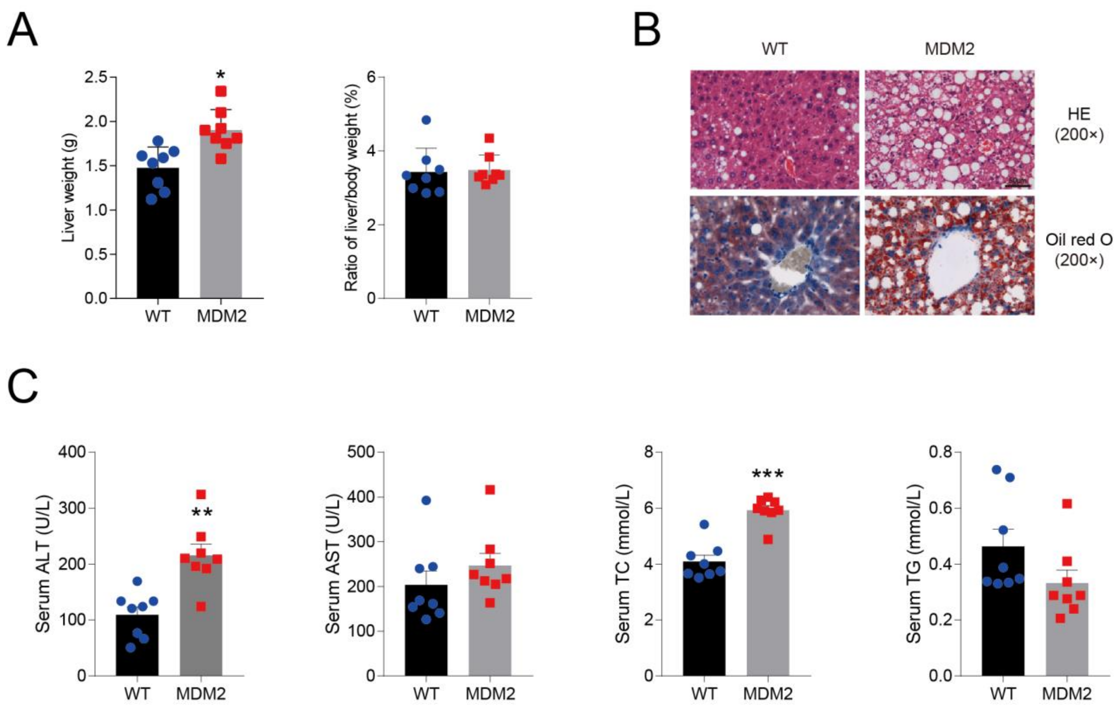

Figure S6. MDM2 overexpression in adipose tissues aggravates HFD-induced hepatic steatosis. Related to Figure 5.

(A) Liver weight (left) and ratio of liver weight/body weight (right) in WT and *Mdm2*-AKI mice on HFD for 8 months. n = 8/group. Data are represented as mean ± SD. Statistical analysis was carried out by Student's t test. * $p < 0.05$.
(B) H&E (up) and oil red O staining (down) of livers of WT and *Mdm2*-AKI mice on a HFD for 8 months. Scare bars, 50 μm.
(C) Biochemical analysis of serum in WT and *Mdm2*-AKI mice on a HFD for 8 months. n = 8/group. Data are represented as mean ± SD. Statistical analysis was carried out by Student's t test. ** $p < 0.01$ and *** $p < 0.001$.

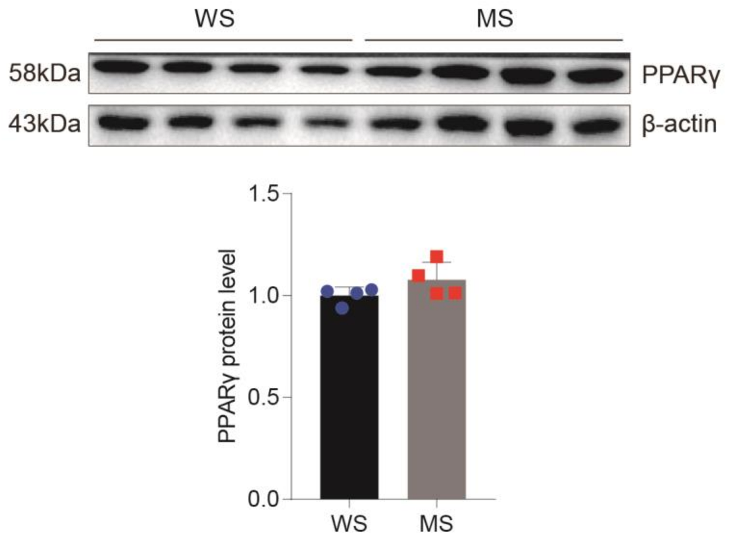

Figure S7. Western blotting (up) and protein gray value (down) of PPARγ in eWAT of WS and MS mice on a HFD for 12 weeks. Related to Figure 8.

n = 4/group. Data are represented as mean ± SD. Statistical analysis was carried out by Student’s t test.

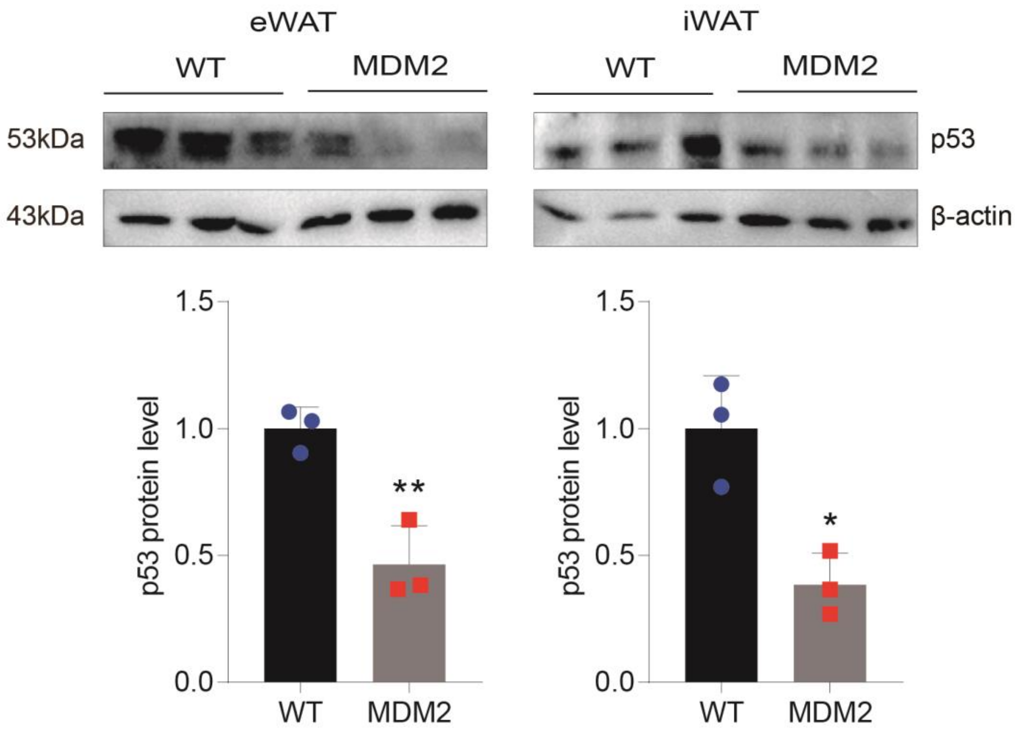

Figure S8. Western blotting (up) and protein gray value (down) of p53 in eWAT (left) and iWAT (right) of WT and *Mdm2*-AKI mice on a HFD for 12 weeks. Related to Figure 7.

n = 3/group. Data are represented as mean ± SD. Statistical analysis was carried out by Student's t test. * $p < 0.05$ and ** $p < 0.01$.

Supplementary Tables

Table S1 Combining analysis proteomics and ubiquitinomics. Related to Figure 6.

Protein accession	Gene name	Downregulation Ratio	Ubiquitin Modified Upregulation Position
Q9D2R0	*Aacs*	0.242	24
Q61285	*Abcd2*	0.619	615
Q9WTQ5	*Akap12*	0.486	164
O70423	*Aoc3*	0.604	596
O54754	*Aox1*	0.641	1103
Q63918	*Cavin2*	0.582	156
Q9DB34	*Chmp2a*	0.639	9
P56198	*Cidec*	0.577	6, 112, 115, 180, 236
P56395	*Cyb5a*	0.57	39
P56387	*Dynlt3*	0.572	63
Q3TGW2	*Eepd1*	0.631	156
P54310	*Lipe*	0.664	323
P26645	*Marcks*	0.472	11, 30, 40
Q61753	*Phgdh*	0.465	21, 384
Q8BJ56	*Pnpla2*	0.594	92
P31324	*Prkar2b*	0.626	174, 202, 285, 370
Q99K85	*Psat1*	0.633	323
Q9ET01	*Pygl*	0.665	618
P07758	*Serpina1a*	0.443	186
P22599	*Serpina1b*	0.517	292
P82347	*Sgcd*	0.554	23
P51912	*Slc1a5*	0.53	7, 384, 502, 534
Q9Z0F7	*Sncg*	0.463	23, 110, 119
Q923B6	*Steap4*	0.658	18, 97, 161

Table S2 Primers of plasmids construction for Co-Immunoprecipitation and In vitro ubiquitination assay. Related to Plasmid construction in STAR Methods.

Plasmids	Forward primer(5'---3')	Reverse primer(5'---3')
pEGFP-C1<br>-STEAP4	ATTCTGCAGTCGACGGTACC<br>GAGAAAGCACATGCA	TAGATCCGGTGGATCCTTAA<br>ATATCCGATT
pCMV3-myc<br>-mSTEAP4	GCCGCCACCAAGCTTGGTA	GAATTCGGCGGCCGCTCTAG
pCMV3-myc<br>-mSTEAP4(K18R)	CTGATTCCTCAGAAAGGCAA<br>GGGGTTGTC	GACAACCCCTTGCCTTTCTG<br>AGGAATCAG
pCMV3-myc<br>-mSTEAP4(K97R)	CTAGTTGATTATCTCAGAGG<br>AAAAGTATTG	CAATACTTTTCCTCTGAGATA<br>ATCAACTAG
pCMV3-myc<br>-mSTEAP4(K161R)	GTGGAAATGACAGCAGGG<br>CCAAACAAAGAG	CTCTTTGTTTGGCCCTGCTG<br>TCATTTCCAC

Table S3 The qPCR primers. Related to Quantitative RT-PCR in STAR Methods.

Gene name	Forward primer (5'---3')	Reverse primer(5'---3')
actin	CCAGCCTTCCTTCTTGGGTAT	TGCTGGAAGGTGGACAGTGAG
Mdm2	CATGTCTGTGTCTACCGAGGG	TAAGTGTCGTTTTGCGCTCC
p21	TAAGGACGTCCCACTTTGCC	GACAACGGCACACTTTGCTC
Bax	GCGTGGTTGCCCTCTTCTACTTTG	AGTCCAGTGTCCAGCCCATGATG
Bcl2	TGAGTACCTGAACCGGCATCT	GCATCCCAGCCTCCGTTAT
Tnfα	CGTCAGCCGATTTGCTATCT	CGGACTCCGCAAAGTCTAAG
Il-6	AGTTGCCTTCTTGGGACTGA	TCCACGATTTCCCAGAGAAC
